# Factors which predict violence victimization in Uganda

**DOI:** 10.11604/pamj.2014.19.335.3480

**Published:** 2014-11-28

**Authors:** Lincoln Fry

**Affiliations:** 1 Sociology Research Unit, Athens Institute for Education and Research (ATINER), Athens, Greece

**Keywords:** Interpersonal violence, property crime, re-victimization, violence prevention, target hardening, fear of crime, Uganda

## Abstract

**Introduction:**

Violence is a major public health issue, globally and on the African continent. This paper looks at Uganda and begins the process of identifying the factors that predict violence in that country. The purpose is to interpret the implications of the study results for violence prevention programs.

**Methods:**

The study includes the responses of 2 399 Ugandans collected in 2011 by the Fifth Round of the Afrobarometer surveys. The study concentrates on 259 respondents who reported either they or someone else in their family had been the victim of violence, defined as being physically attacked, in the last year.

**Results:**

Logistical regression analysis identified six factors that predict physical violence in Uganda. In order, these included being the victim of a property crime, age, gender, fear of crime in the home, poverty, and residential crowding. The surprising findings relate to what may be called target hardening, especially for those likely to be re-victimized. Respondents did tend to be re-victimized, with about 61 percent of violence victims also property crime victims. Fear of crime in home was another predictor of violence victimization, and many of these respondents had been crime victims.

**Conclusion:**

These findings imply that target hardening should be the basis to begin to implement violence prevention programs in Uganda. The suggestion is crime prevention personnel/ law enforcement need to respond to reported incidents of property and/or violence victimization and attempt to prepare victims to protect both their premises and their persons in the future.

## Introduction

In 1996, the World Health Organization (WHO) declared violence a major public health problem [1]. In 2000, WHO created the Department for Injuries and Violence Prevention [2], and in 2002, released the World Report on Violence and Health [3]. Violence was included in the call for improved research that highlighted public health's need to address data collection deficiencies, including hospital and police records, in order to begin to develop preventive interventions, including injury control programs. Violence is a major societal problem in Uganda, which is rated 7^th^ worst in the world in violence deaths, and violence as the cause of death ranks 9^th^ in the country [4]. The majority of the research concerned with violence in Uganda has most recently concentrated on HIV, [5] domestic violence [6] and youth violence [7] as well as the effects of civil war [8].

There has been an increasing volume of calls to develop violence prevention programs at the country, continental and international levels, as well as the concomitant need to begin to develop violence prevention programs [9]. One approach which has gained some support in Africa, and elsewhere is called target hardening and is derived from what is known as the built environment framework [10]. Elements in the built environment include homes, schools, workplaces, parks/recreation areas, business areas and roads. It encompasses all buildings, spaces and products that are created or modified by people. This approach endorses a crime prevention approach called CPTED (Crime Prevention Through Environmental Design), and target hardening falls under that rubric. Research in this tradition has focused mainly on housing, transportation and neighborhood characteristics [11], emphasizing improved protection of self, Property and neighborhoods [12], as well as areas like counties in the US [13]. Inadequate urban planning has been identified as a major source of problems in those areas, and some studies indicate that the impact of mediating and moderating factors within the built environment must be the focus of future health research [14]. There are issues that have been raised about CEPTED and Public Health strategies and they will be addressed in the conclusion. Uganda has been included in several international crime surveys. One was sponsored by the United Nations Office on Drugs and Crime... Conducted in 2007 [15], the findings showed that 14 percent of respondent households had been victimized in 2007, with 8 percent burglary victims and 9 percent victims of attempted burglary. Fear of crime measures revealed that 23 percent felt very unsafe walking alone in their area, 13.6 percent felt very unsafe home alone after dark and 11.1 percent felt the chances were very likely someone would break into their homes in the next 12 months.

The International Crime Victim Survey (ICVS) [16] was first conducted in 1998, again in 2000, and most recently in 2006. The survey's study population includes all adult Ugandan males and females 18 years of age and older. Data collection consists of face-to-face personal interviews utilizing a stratified multi-stage representative sample random selection process designed to generate a nationally representative sample. Respondents are asked about their crime experiences during the prior calendar year as well as over the last five years. The important findings from the 2006 report regarding Uganda were as follows: 1) reported one of the lower assault rates (26.6 percent); 2) reported the third highest threat rate (64.1 percent) and; 3) was found to have one of the lower household burglary rates, (5.9 percent). Uganda did report one of the highest rates of ownership of special locks, and high levels of membership in formal neighborhood watch programs, 43.4 percent. One of the report's conclusions was that multiple victimizations are a major problem in Africa and crime victimization studies therefore do not reflect true victimization rates.

## Methods

This study's Data Source is Afrobarometer, a collaborative research effort produced by social scientists from 35 African countries. The Project′s objectives are as follows; 1) to produce scientifically reliable data on public opinion in sub-Saharan Africa; 2) to strengthen institutional capacity for survey research in Africa; and 3) to broadly disseminate and apply survey results. Begun in 1999, five rounds of the survey have been completed; Uganda was included in all five waves, as well as two other country specific surveys. The most recent survey was conducted in 2011, and became available in 2013.

The Survey consisted of face-to face Interviews completed by 2 399, 18 years of age or older. These interviews were conducted in fifteen different languages. The sampling frame included all nine Ugandan provinces, and the final sample supports estimates to the national population of all adults in Uganda that is accurate to within a margin of error of plus or minus 2 percentage points at a confidence level of 95 percent. The sampling procedures used in all of the Afrobarometer surveys are explained in detail in Bratton, Mattes and Gyimah-Boadi (2005) [17].

**The dependent variable:** violence victimization: survey respondents were asked about criminal victimization. One question asked “over the past year, how often, if ever, have you or anyone in your family been physically attacked?” Fixed responses were provided as follows: never; just once or twice; several times; many times; and always. The study's dependent variable was created by treating never as one category (0) and all other affirmative responses were coded as one (1). This dichotomous variable is the study's dependent variable and provides the basis for the logistic regression presented below.

**The independent variables:** a poverty scale used in the Afrobarometer studies was adopted from Mattes et al. (2003) [18], factor scaled, scale scores were calculated and assigned to each respondent; The Questions which generated the scale were “over the past year, how often, if ever, have you or anyone in your family gone without the following”; enough food to eat, enough clean water for home use, without medical care, enough fuel to cook your food and, a cash income? This scale's reliability Coefficient was 83 (Cronback's Alpha). The control variables listed in Table 1 were measured by a single item, like age, and others were collapsed into fewer categories; for instance, race, which became a dichotomous variable, Black Africans and all others, and education, which was reduced to five categories, by combining no school, informal, only and some primary. Other variables were also measured by single items, including the fear of crime in the home and neighborhood, property crime victimization and trust of the police. Others, like the presence of a police station in the respondent's local area, whether police were visible in the local area, and residential crowding were recorded by the interviewer andsupplemented/checked by the interviewer's supervisor.

**Table 1:** Demographic characteristics of the Ugandan respondents by violence victimization (N = 2 399)

Victim of Violent crime				
Variable	Yes	No	Total	P. value
**Age**				
18 through 29	146 (15.4)	805 (84.6)	951	0.39
30 through 49	107 (11.3)	841 (88.7)	948	
50 and older	33 (5.6)	556 (94.4)	589	
**Gender**				
Male	155 (12.9)	1044(87.1)	1199	0.000
female	104 (8.7)	1096 (91.3)	1200	
**Ethnicity**				
Black african	194 (12.6)	1340 (87.4)	1534	0.000
Coloured/mixed race	31 (9.0)	315 (91)	346	
White/ European	15 (3.6)	398 (96.4)	413	
South/East Asians/Arabs	19 (18.1)	86 (81.9)	105	
**Religion**				
Christain	224 (11.2)	1781 (88.2)	2005	0.54
Muslim	61 (33.2)	123 (66.8)	184	
None/all others	15 ( 8.5)	162 (91.5)	177	
**Education**				
No formal/informalschooling	78 (27.8)	203 (72.8)	281	0.000
Some/primaryschoolcompleted	181(17.8)	839 (82.2)	1020	
Some/completedhigschoo	113(14.0)	603 (86.0)	806	
Post-secondary qualification	19(9.9)	173(90.1)	192	
Completed university	15 (16.9)	79 (84.0)	94	
Graduate school				
**Residence**				
Urban	42 (12.2)	302 (87.8)	344	0.01
Rural	365 (17.8)	1690 (82.2)	2055	
**Employment**				
Unemployed	253 (20.4)	987 (79.6)	1240	0.000
Employed part-time	91(16.4)	465 (83.6)	556	
Employed full time	62 (10.3)	539 (89.7)	601	

## Results

The sample social and demographic characteristics are displayed in Table 1, broken-down by whether respondents were or were not victims of physical violence within the last year.

Table 1 shows that there was a statistically significant difference in violence victimization by age, gender and ethnicity in this Ugandan sample. Younger respondents were more likely to be victimized and males were also more likely to be violence victims than were females. Black Africans and South/ East Asians/Arabs were more likely than coloured/mixed race persons to be victimized, with White Europeans least likely; all of those significance levels were at the .001 level or higher. There was no significant difference in violence victimization by faith, educational level, or rural, as opposed to urban residents and employment status.

In Table 2, violence victimization in the last year is displayed for selected independent variables. These items begin with fear of crime in the home and in the neighborhood,. and include crowding, and the number of adults living in each residence. The other measures were those observed by the interviewer and verified by the field supervisor. Table 2 shows that fear of crime and in the home and neighborhood were found to be significant independent variables related to violence victimization. Both fear of crime measures were significant at the .000 level. Of those who reported that they were afraid of crime in the home, 197 had been violent crime victims, as had 196 respondents who reported they were fearful about crime in the neighborhood; these findings are addressed further below.

**Table 2:** Cross-tabulation violence victimization and selected independent variables

Victim of Violent Crime				
Variable	Yes	NO	Total	P. value
**Fear of crime-home**				
Yes	197 (16.1)	1024 (83.9)	1221	0.000
No	61 (5.2)	1113 (94.8)	1174	
**Fear of Crime-neighborhood**				
Yes	196 (14.3)	1174 (85.7)	1370	0.000
**No**	**63 (6.1)**	**963 (93.9)**	**1026**	
**Residential Crowding**				
One or two adults	89 (9.4)	858 (90.6)	947	0.14
Three or four adults	126 (11.3)	987 (88.7)	1113	
Five or more adults	39 (13.1)	258 (86.9)	297	
**Police station in area**				
Yes	120 (9.9)	1092 (90.1)	1212	0.16
No	138 (11.7)	1041 ( 88.3)	1179	
**Police Visible in area**				
Yes	113(10.1)	1003 (89.9)	1116	0.32
**No**	**146 (11.4)**	**1137 (88.2)**	**1283**	
**Trust the police**				
Yes	206 (10.4)	1773 (89.6)	1979	0.09
No	53 (13.3)	346 (86.7)	399	
**Health Clinic in the area**				
Yes	157 (10.2)	1378 ( 89.8)	1535	0.19
No	102 (12.0)	750 (88.0)		

The independent variables listed in Table 1 and Table 2 were included in the logistical regression presented in Table 3, with violence victimization the dependent variable.

**Table 3:** Logistic regression with violence victimization as the dependent variable

Variable	Coefficient	Standard Error	Z	P-value
**Property crime victim**	**1.51**	**0.149**	**10.16**	**0.00**
Age	-0.412	0.103	-3.99	0.00
Gender	-0.58	0.150	-3.87	0.00
Fear of crime -home	0.21	0.057	3.54	0.00
Poverty	0.06	0.018	3.60	0.00
Residential crowding	0.064	0.033	1.93	0.05
**Employment**	**0.090**	**0.900**	**1.01**	**0.31**
**Faith**	**-0.000**	**0.000**	**-1.02**	**0.31**
Health clinic in area	-0.22	0.191	-1.14	0.25
Urban rural	0.014	0.17	0.08	0.93
Trust police	0.13	0.184	0.07	0.96
Police station in area	0.11	0.200	0.57	0.57
Police visible	-0.145	0.170	-0.86	0.39
Education	-0.038	0.091	-0.42	0.49
Ethnicity	-0.03	0.090	-0.29	0.77
Constant	2.48	0.700	3.85	0.00
Number of observations =2 207				
**Chi square = 241.81**				
**Probability = 0.000**				
**Pseudo R2 = 0.16**				

Table 3 reveals that six independent variables reached significance in the logistical regression analysis. Five of these were highly significant, with property crime victimization the strongest, z = 9.88. The poverty measure was next, z = 4.06, followed in order by age, z = -3.99, gender, z = 3.77 fear of crime, z = 3.56. All of the other independent variables reached the .000 level of significance. Residential crowding was also significant, z = 2.04, p = =.04.. None of the other variables in Table 3 reached significance. The regression results produced a pseudo R2 of 16.

The surprising finding in Table 3 was the strength of the property crime victimization measure in the regression equation. As a result, Table 4 takes a closer look at the violence and property crime measures.

**Table 4:** Crosstabulation of property and violent crime victimization

Victim of Violent Crime			
Variable	Yes	NO	Total
**Victim of Property Crime**			
Yes	157(25.7)	454 (74.3)	611
No	102 (5.7)	1686 (94,3)	1788
**Total**	**259**	**2140**	**2399**

Chi-Square = 188.97 P value =0.000

Table 4 reveals that 713 persons, 29.7 percent of these Ugandan respondents, had been crime victims within the last year. Of these, 157 of 259 the identified violence victims were also victims of property crimes (60.6 percent). This fact points to the need to start thinking about the multiple/re-victimization of these Ugandan respondents... Note that in Table 2 there were 216 respondents that indicated they had fear of crime who were also victims of violent crime.

## Discussion

Before the implications of these findings for crime prevention in Uganda are addressed, there are several issues that need to be mentioned. One of these is what Shepard [19] defined as criminal deterrence as a public health strategy. As Shepard suggested, despite the fact that violence is now seen as a public health issue, criminal deterrence as a public health strategy has been greeted with ambivalence and even hostility. That reality needs to be addressed and clearly reassessed. A second issue is methodological. The results of the findings presented in Table 2 and Table 4 point to a weakness in this study, and highlights a requirement for future research. This is the need to establish the time priority for the physical and property crime victimization. We are unable to determine from this data which victimization occurred first or if they occurred at the same point in time; that is the old problem that correlation does not necessarily mean causation. This same caution applies to the fear of crime indicators. In terms of fear of crime, the question is whether these respondents did have a valid reason to fear crime, because a large percentage of them had in fact been victims of crime.

## Conclusion

The logistical regression analysis showed that there were five highly significant factors that predicted violence in Uganda. In order of their magnitude, these included being a victim of property crime, poverty, age, gender, and fear of crime in the home; residential crowding also reached significance in the logistical regression analysis, at a lesser significance level. These findings present possible ways to approach crime prevention programs in Uganda. They suggest that target hardening should be the basis to begin to develop and implement violence prevention programs. This approach would mean that crime prevention/ law enforcement personnel should respond and follow-up incidents of reported property and/ or violence victimization in their jurisdictions. The purpose would be to attempt to prepare and assist victims to better protect both their premises and their persons. Target hardening refers to issues like improving locks, installing proper night lighting and clearing bushes from in front of their windows that might impede visibility of their property and neighborhoods. Personal experience with target hardening programs suggests that residents become open to target hardening approaches, and personnel, once they have been victimized. Also, once victimized, residents are more encouraged to develop local neighborhood groups that provide security for themselves and those in their own communities.

## References

[1] World Health Assembly (1996). Prevention of violence: public health priority (WHA 49,25).

[2] Krug E, Sharma Glozanno (2000). The Global Burden of Injuries: commentaries. Am J Public Health.

[3] Krug EG, Dahlberg LL, Mercy JA, Zwi AB, Lozano R (2002). World Report on violence and health.

[4] World Health Rankings (2013). Violence. http://www.worldlifeexpectancy.com/ugandsviolence.

[5] Kuznic A, Lamonde M, Hermans S, Casteinuovo B, Auerbach B, Semeere A (2012). Evaluating the cost-effectiveness of combination antiretroviral therapy for the prevention of mother-to-child transmission of HIV in Uganda. Bull World Health Organ.

[6] Swahn M, Gressard L, Palmer C, Kasirirye R, Lynch C, Yao M (2012). Serious violence Victimization and Perpetration among Youth Living in the Slums of Kampala, Uganda. Western Journal of Emergency Medicine.

[7] Wagman J, Namatovu F, Nalugoda F, Kiwanuka D, Nakigozi G, Gray R (2012). A public Health Approach to Intimate partner Violence Prevention in Uganda. Violence Against Women.

[8] DeLuca G, Verpoorten M (2012). From Vice To Virtue? Civil war and Social Capital in Uganda.

[9] Srinivasan S, O'Fallon L, Dearyy A (2003). Creating Healthy Communities, Healthy Homes, Healthy People: Initiating a Research Agenda on the Built Environment andPublic Health. American Journal of Public Health.

[10] Rapoport A (1982). The Meaning of the Built Environment: A Nonverbal Communications Approach.

[11] Ahianba J, Dimuna K, Okogun G (2008). Built Environment Decay and Urban Health in Nigeria. J Human Ecology.

[12] Sampson R, Raudenbusn S, Earls F (1997). Neighborhoods and Violent Crime: A Multilevel Study of Collective Efficacy. Science.

[13] Harries K (2008). Property Crimes and Violence in United States: An Analysis of the influence of Population density. International Journal of Criminal Justice Sciences.

[14] Cozens P (2007). Public health and the potential benefits of Crime Prevention Through Environmental Design. New South Wales Public Health Bulletin.

[15] Naude C, Prinsloo J, Ladikos A (2006). Experiences of Crime in Thirteen African Countries.

[16] Results from The International Crime Victim Survey (2006).

[17] Bratton Michael, Robert Mattes, Gyimah-Boadi E (2005). Public Opinion, Democracy, and Market Reform in Africa.

[18] Mattes R, Bratton M, Davids Y (2003). Poverty, Survival, and Democracy in Southern Africa, Afrobarometer.

[19] Shepard J (2001). Criminal deterrence as a public health strategy. Lancet.

